# Sickle Cell Disease and Antimicrobial Resistance: A Systematic Review and Meta-Analysis

**DOI:** 10.3390/idr17020032

**Published:** 2025-04-14

**Authors:** Bismark Opoku-Asare, Onyansaniba K. Ntim, Aaron Awere-Duodu, Eric S. Donkor

**Affiliations:** 1 Department of Medical Microbiology, University of Ghana Medical School, Accra P.O. Box KB 4236, Ghana; kuspusbizi2@gmail.com (B.O.-A.); okntim001@st.ug.edu.gh (O.K.N.); aawereduodu@gmail.com (A.A.-D.); 2 Infectious Disease Center, Department of Medicine and Therapeutics, Korle Bu Teaching Hospital, Accra P.O. Box KB 4236, Ghana

**Keywords:** AMR, antibiotic resistance, sickle cell disease, bacteria

## Abstract

**Background/Objectives**: Antimicrobial resistance (AMR) is increasingly rising due to antimicrobial overuse and misuse. In sickle cell disease (SCD) care, frequent antibiotic use drives the rapid emergence of AMR, threatening treatment options and patient lives. This systematic review synthesizes data on AMR with regard to SCD patients for the first time. **Methods**: A comprehensive database search for articles published in English was conducted in PubMed, Scopus, ScienceDirect, and Web of Science, with no restriction set for the year of publication. The DerSimonian–Laird method was applied to derive the pooled prevalence, while the Mantel–Haenszel method was used to calculate the pooled odds ratio. **Results**: A total of 18 eligible studies covering 3220 SCD patients published between 1996 and 2024 were included in this review. The common bacterial pathogens reported in the included studies were *Streptococcus pneumoniae* (10 studies), *Staphylococcus aureus* (10 studies), and *Escherichia coli* (4 studies). For *S. aureus*, the pooled resistance was highest for penicillins (ampicillin = 100%; penicillin = 93.64%; and amoxicillin = 77.82%) followed by cefuroxime (51.23%). The pooled prevalence of methicillin-resistant *S. aureus* (MRSA) was 19.30%. SCD patients had 2.89 and 2.47 times higher odds of being colonized or infected with penicillin-resistant and erythromycin-resistant *S. aureus* strains, respectively. For *S. pneumoniae*, resistance prevalence was highest for co-trimoxazole (81.1%), followed by penicillin (47.08%). The pooled prevalence of multidrug-resistant (MDR) *S. pneumoniae* isolates was 32.12%. The majority of the studies included (*n* = 14, 77.8%) were of moderate quality according to the modified STROBE checklist. **Conclusions**: This review reveals a high prevalence of AMR with regard to SCD patients. SCD patients have an increased risk of resistance to penicillin and co-trimoxazole across several bacterial pathogens. The limited geographical distribution of the included studies underscores the urgent need for expanded AMR research on the subject, especially in regions with high SCD burden.

## 1. Introduction

The discovery of antibiotics in the 20th century revolutionized the treatment of bacterial infections, saving millions of lives. However, the subsequent overexploitation and misuse of these life-saving drugs have contributed to the development of antimicrobial resistance (AMR) [1,2]. This “silent pandemic” has gained alarming attention in recent decades, with the World Health Organization (WHO) recognizing AMR as a major global health threat. Inadequate enforcement and non-compliance to guidelines, policies, and regulations regarding antimicrobial use in humans and animals have significantly accelerated the emergence and spread of resistant pathogens [3]. The rise of antimicrobial resistance is increasingly limiting the available treatment options for infections, resulting in millions of deaths these past few years. Bacterial antimicrobial resistance, the most common type of AMR, directly caused 1.27 million deaths and contributed to more than 4 million deaths globally in 2019 [4]. Vulnerable populations, including those with sickle cell disease (SCD), face exacerbated risk of severe outcomes from antimicrobial-resistant infections [5,6].

Sickle cell disease (SCD) is a group of inherited genetic disorders affecting the hemoglobin molecule in the red blood cells, causing cells to assume a sickle-like shape [7,8]. The most common and clinically important genotype of sickle cell disease is homozygous SCD, known as sickle cell anemia (HbSS). Other rare forms of SCD include sickle cell beta-thalassemia (HbS/β-thalassemia), sickle cell HbC (HbSC) disease, hemoglobin SD, and hemoglobin SE. Approximately, 5% of the world’s population globally carries the genes responsible for SCD, affecting more than 250,000 live births yearly [9]. The disease is prevalent in malaria-endemic regions in Africa, the Middle East, the Caribbean, and South Asia [10,11,12]. SCD is a substantial global health concern, with an estimated 7.74 million people living with the condition, and 376,000 deaths reported worldwide in 2021 [11].

The abnormal shape of the red blood cells compromises the immune system of SCD patients, putting them at risk of severe health complications, including frequent severe pain crises, asplenia, severe infections, stroke, severe anemia, and an increased mortality risk [8]. Infections, particularly invasive bacterial infections, are the leading cause of morbidities and mortalities in SCD patients [13,14]. Frequent hospitalization, antibiotic therapy, and invasive medical procedures predispose SCD patients to infection with various resistant bacterial pathogens [15,16]. The convergence of SCD-related immunocompromization and the escalating issue of AMR creates a perfect storm of vulnerability, limiting treatment options and putting SCD patients at risk of severe outcomes. Despite the wealth of research data on AMR in pathogens isolated from SCD patients, a systematic review to comprehensively assess the global prevalence of AMR in bacterial pathogens isolated from SCD patients is still lacking. This systematic review aims to fill this knowledge gap by providing a global analysis of the antibiotic resistance patterns of these pathogens. Our analysis will shed light on the prevalence, patterns, and factors associated with AMR in this vulnerable population, informing evidence-based strategies to mitigate the AMR threat and improve health outcomes for SCD patients worldwide.

## 2. Materials and Methods

### 2.1. Database Search Strategy

This systematic review followed the Preferred Reporting Items for Systematic Reviews and Meta-Analyses (PRISMA) guidelines (Supplementary file_PRISMA_checklist_SCDandAMR2024) [17]. A comprehensive literature search was conducted across four known databases—PubMed, Scopus, ScienceDirect, and Web of Science—using a predefined search strategy. The search keywords combined the keywords “Antimicrobial Resistance” AND “Bacteria” AND “Sickle Cell Disease” to retrieve relevant studies. The search was restricted to full-text, peer-reviewed research articles published in English, with no restriction set for publication date. The search results were imported into Rayyan online (https://www.rayyan.ai/, accessed on 13 August 2024) [18], a web-based systematic review management tool. A detailed description of the search strategy, including database-specific keywords and filters, is provided in Table S1 of the Supplementary Material.

### 2.2. Study Selection Criteria

Two independent reviewers screened the titles, abstracts, and full texts of the remaining articles after duplicate removal against the predetermined eligibility criteria. Studies were included if they described antimicrobial resistance patterns in bacterial pathogens isolated from infection or colonization in SCD patients and stratified results by bacterial species and antimicrobials. Studies that reported multidrug-resistant bacteria in SCD patients were included. Studies were also included if resistance was described for related bacterial species (such as *E. coli* and *K. pneumoniae* from the *Enterobacteriaceae* family). Case studies, reviews, brief reports, commentaries, and studies lacking specific data on AMR in sickle cell disease patients were excluded. The outcomes of the two reviewers were compared, and any disagreements were resolved through consensus discussion.

### 2.3. Data Extraction

Data from the included studies were extracted in Microsoft Excel 365 Version 2108 (Microsoft, Redmond, WA, USA) by two reviewers. The extracted data comprised various study characteristics, including author and year of publication, country, study design, year of study, age group, study population, number of participants, isolate type, isolate site, organism, method of antimicrobial susceptibility testing, and patient characteristics. For each bacterial pathogen, the number of isolates resistant to a particular antimicrobial was also extracted. The Freeman–Tukey double arcsine transformation was used in stabilizing variances among studies.

### 2.4. Data Analysis

RStudio version 4.3.3 (Posit PBC, Boston, MA, USA) was used to perform a single-group prevalence meta-analysis and an odds ratio meta-analysis, employing the metaprop and metabin functions, respectively. The DerSimonian–Laird method was applied to derive the pooled prevalence, while the Mantel–Haenszel method was used to calculate the pooled odds ratio. Heterogeneity was assessed using the I^2^ statistic, with ≥25%, 50%, and ≥75% interpreted as low, moderate, and high heterogeneity, respectively. Statistical significance was set at a *p*-value of <0.05.

### 2.5. Quality Assessment

Two investigators assessed the quality of the included studies using the STROBE checklist for reporting observational studies. The checklist was modified to include the following 15 items: study background and rationale, objectives, design, setting, eligibility criteria, variable definition, data source, bias, study size, statistical method, included participants, descriptive data, outcome data, key results and interpretation, and limitations. Each item was either answered ‘YES’ if the study provided adequate information or ‘NO’ if the study had no information or an unclear description. Studies were graded into three categories: high quality (yes for 11–15 items), moderate quality (yes for 6–10 items), and low quality (yes for 1–5 items).

## 3. Results

### 3.1. Study Selection Process

**Figure 1:**
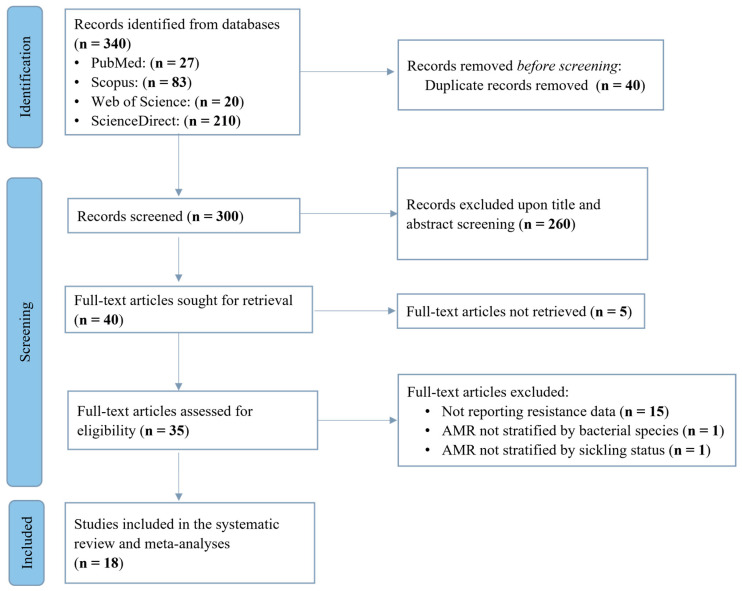
Prisma flow diagram of study search and selection process.

The database search retrieved 340 records, from which 40 duplicates were removed. The remaining 300 unique records underwent title and abstract screening, resulting in 40 full-text articles being assessed for eligibility. Following full-text assessment, 22 articles did not meet the study’s eligibility criteria. Finally, a total of 18 articles were included in the analysis (Figure 1).

### 3.2. Characteristics of Included Studies

**Figure 2:**
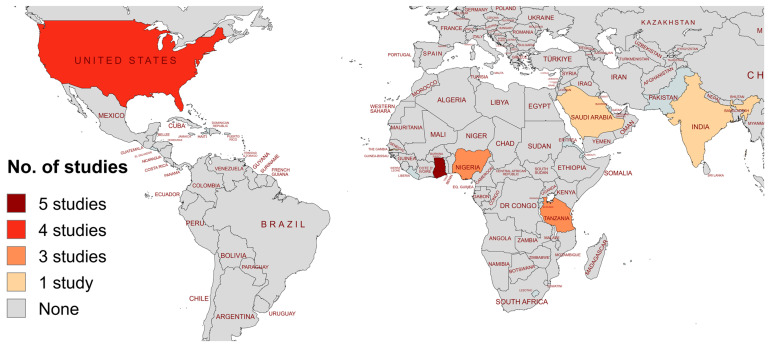
Geographical distribution of included studies.

The included studies were conducted in 7 countries, of which half of them were either from Ghana (*n* = 5, 27.7%) or the United States (*n* = 4, 22.2%). More than half of the studies were conducted in low- and middle-income countries (*n* = 13, 72.2%) (Figure 2). The eligible studies included 3220 SCD patients. Four (55.6%) studies included HbSS patients only, two (11.1%) studies included both HbSS and HbSC patients, two (11.1%) studies included HbSS, HbSC, and HbSβ-thalassemia patients, and ten (55.6%) studies did not specify the sickling genotype of the patients included. Only three (16.7%) studies reported the method for sickle cell diagnosis. Seven out of the 18 studies included control groups (HbAA) with a total of 987 individuals. The majority of the studies reported data from children (*n* = 12, 66.7%), while two (11.1%) included only adults and three (16.7%) included both children and adults. Out of eight studies presenting data for nasopharyngeal carriage, four (50%) studies reported patients with respiratory symptoms, and three (37.5%) studies each described patients with asthma and pneumonia. Fever was reported in three out of four studies describing SCD patients with urinary tract infections. Patients taking penicillin prophylaxis were reported in seven studies (38.9%). All the patient characteristics have been summarized in Table S2 in the Supplementary Material.

Most studies reported resistance data for a single pathogen (*n* = 11, 61.1%), while eight studies described data for multiple pathogens (Table 1). Of the 18 studies, 10 studies (55.6%) described resistance in bacterial carriage/colonization (nasopharyngeal, *n* = 7; nasal only, *n* = 1; nasal and nasopharyngeal, *n* = 1; and rectal, *n* = 1). Eight studies, on the other hand, focused on bacterial infections, with most studies presenting resistance data for pathogens from either the urine (*n* = 4, 22.2%) or blood (*n* = 3, 16.7%). AMR in *Staphylococcus aureus* and *Streptococcus pneumoniae* was presented in ten studies each, and *Staphylococcus* spp. and *Streptococcus* spp. in one study each. Studies describing resistance in Enterobacteriaceae were on *Escherichia coli* (*n* = 4), *Klebsiella* spp. (*n* = 3), *Klebsiella pneumoniae* (*n* = 1), *Pseudomonas aeruginosa* (*n* = 1), *Pseudomonas* spp. (*n* = 2), *Salmonellae* spp. (*n* = 2), *Proteus* spp. (*n* = 2), *Acinetobacter baumannii* (*n* = 1), Coliform (*n* = 1), and *Enterobacteriaceae* (*n* = 1). The disc diffusion method for antibiotic sensitivity testing (AST) was widely used in the majority of the included studies (*n* = 13, 72.2%). Other methods utilized included the E-test (three studies), broth microdilution (one study), and VITEK 2 system (one study). The description of studies is summarized in Table 1.

### 3.3. Antibiotic Resistance in Staphylococcus aureus

Antimicrobial resistance in *S. aureus* was described in 10 studies. Five studies focused on *S. aureus* infection (urinary tract infection *n* = 3; blood *n* = 2). The highest pooled resistance among *S. aureus* isolates infecting SCD patients was estimated for penicillins (penicillin = 99.99%, 95% CI [94.87; 100.00]; ampicillin = 98.15% 95% CI [49.83; 100.00]; and amoxicillin = 77.82%, 95% CI [61.93; 91.16]) while the lowest pooled resistance was estimated for fluoroquinolones (ciprofloxacin = 16.10% 95% CI [7.03; 27.31]). Heterogeneity was high across studies reporting resistance to ampicillin, co-trimoxazole, and erythromycin. Moderate heterogeneity was observed across studies for cefuroxime and gentamicin (Table 2 and Figure S1).

**Figure 3:**
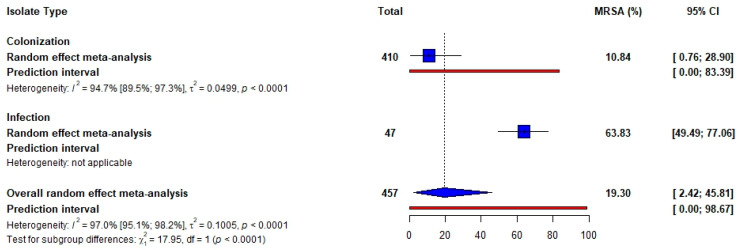
Pooled prevalence of Methicillin-resistant *Staphylococcus aureus* isolated from SCD patients.

*S. aureus* carriage was described in five studies (nasopharynx *n* = 3; nasal *n* = 1; and nasopharynx and nasal *n* = 1). The pooled prevalence was estimated to be highest for penicillin (90.47%, 95% CI [57.19; 100.00]) and lowest for clindamycin (11.02%, 95% CI [2.08; 25.13]). High heterogeneity was observed across all studies (Table 2 and Figure S2). Half of the studies (*n* = 5) identified methicillin-resistant *S. aureus* (MRSA) isolates. The pooled prevalence of four studies describing MRSA colonization was 10.84% (95% CI [0.76; 28.90]). The heterogeneity was high across the studies (Figure 3).

SCD patients had 7.62 (95% CI [0.37; 155.87]) times higher odds of penicillin-resistant and ampicillin-resistant *S. aureus*. The pooled OR for erythromycin resistance was 2.30 (95% CI [0.73; 7.18]) among isolates from infection and 2.64 (95% CI [0.87; 8.02]) among isolates from colonization. Heterogeneity was zero across studies for co-trimoxazole (infection) and erythromycin (infection and colonization). Moderate and high heterogeneity was observed across studies for co-trimoxazole (colonization) and amoxicillin (infection) resistance (Table 3).

### 3.4. Antibiotic Resistance in Streptococcus pneumoniae

**Figure 4:**
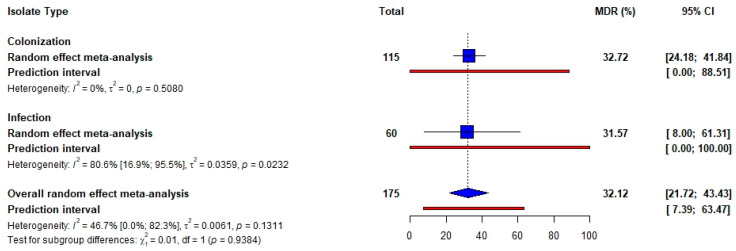
Pooled prevalence of MDR *Streptococcus pneumonia* isolated from SCD patients.

Ten studies reported AMR in *S. pneumoniae*, with the majority focusing on pneumococcal carriage (*n* = 6, 60%). Four studies (40%) described pneumococcal diseases among SCD patients. The pooled resistance was highest for co-trimoxazole (infection = 74.26%, 95% CI [7.14; 100.00] and colonization = 84.99%, 95% CI [70.32; 95.75]) and lowest for erythromycin (infection = 6.50%, 95% CI [0.00; 49.50] and colonization = 18.75%, 95% CI [6.01; 35.48]). Heterogeneity was moderate across studies for penicillin resistance (infection and colonization) and low across studies for erythromycin resistance (infection and colonization). Studies for co-trimoxazole resistance exhibited high and moderate heterogeneity for colonization and infection, respectively (Table 2). The forest plots showing the pooled resistance of *S. pneumoniae* isolated from SCD patients are represented in Figure S3 (Infection) and Figure S4 (Colonization). The pooled prevalence of multidrug-resistant *S. pneumoniae* was 32.2% (95% CI [24.18; 41.84]) for colonization and 31.57% (95% CI [8.00; 61.31]) for infection (Figure 4).

### 3.5. Antibiotic Resistance in Escherichia coli

All studies (*n* = 4) reporting resistance in *E. coli* focused on urinary tract infections. The pooled resistance was highest for ampicillin (96.61%, 95% CI [88.70; 100]) followed by co-trimoxazole (93.17%, 95% CI [72.10; 100]). Cephalosporins had the lowest pooled resistance (ceftriaxone = 24.89%, 95% CI [5.94; 49.18] and cefuroxime = 30.43%, 95% CI [11.52; 52.5]). Heterogeneity was high across studies for co-trimoxazole resistance and low across studies for ceftriaxone resistance (Table 2 and Figure S5).

### 3.6. Quality of the Included Studies

The majority of the studies included (*n* = 14, 77.8%) were of moderate quality according to the modified STROBE checklist (Table S3). The following items were not often discussed: how the study sample size was derived, the outcome data, potential effort to address biases, and variable definitions. The reporting completeness was high in only four studies (22.2%).

## 4. Discussion

Patients with sickle cell disease are particularly prone to severe health complications, leading to significantly increased healthcare utilization, including frequent hospitalizations, which in turn exacerbates their risk of infections, especially bacterial infections, due to invasive medical procedures. As a result of their compromised immune systems, SCD patients rely on antibiotics to combat bacterial infections. However, frequent antibiotic use contributes significantly to the emergence of antibiotic resistance, as bacteria develop resistance when repeatedly exposed to high levels of antibiotics. This study presents novel data on antibiotic resistance among bacterial pathogens isolated from SCD patients, shedding light on this critical public health concern.

Effective treatment of bacterial infections in this era of antimicrobial resistance requires evidence-based knowledge of the susceptibility and resistance of specific bacterial pathogens to a wide range of antimicrobial drugs. Antimicrobial susceptibility testing (AST) assays are crucial in obtaining this information. In our study, we found studies utilizing various types of AST methods, including disc diffusion, broth microdilution (BMD), the VITEK 2 automated system, and the E-test, to investigate the antibiotic susceptibility of isolated bacterial pathogens. Most studies utilized the disc diffusion methods of AST to report resistance data. This method is simple and inexpensive, making it a routinely used AST method in many clinical microbiological laboratories [34,35]. Although the broth microdilution (BMD) method is considered the gold standard for determining an antibiotic’s minimum inhibition concentration (MIC), it requires more time and resources than the disc diffusion method [36,37]. To resolve this limitation, the VITEK 2 system was developed to provide an automated approach to performing the BMD technique, reducing the hands-on time and enhancing workflow, with rapid reporting of the AST results [34,38,39]. However, this system is expensive, and not all clinical laboratories can afford it, limiting its use. The E-test offers a simple approach to determining the MIC of an antibiotic on an agar medium. Nevertheless, it is more expensive than disc diffusion, and its use is restricted to antibiotics available as strips [34,39].

Penicillin is widely used as an antibiotic prophylaxis given to SCD children under the age of five years [40]. However, this may contribute to the increased risk of colonization or infection with antibiotic-resistant pathogens. *S. pneumoniae* is a major cause of invasive bacterial infections such as pneumonia and meningitis [41]. The pooled prevalence of penicillin resistance of 46.61% in *S. pneumoniae* infection exceeded the 29% pooled prevalence in patients with HIV patients [42]. High resistance rates to penicillins were observed among *S. aureus* for both infection and colonization. The rates of penicillin-resistant isolates colonizing and infecting SCD patients were similar. Commensal pathogens like *S. aureus* and *S. pneumoniae* are more susceptible to antibiotic exposure and likely to develop resistance at the colonization stage even before transitioning to cause infection. This may explain the similar pooled penicillin resistance observed during colonization and infection. Our results show that SCD patients had 7.62 times higher odds of carrying penicillin-resistant *S. aureus* isolates compared to individuals without SCD. The widespread use of penicillin as prophylaxis and treatment in SCD patients likely contributes to the high resistance observed in these pathogens. The significant rate of resistance observed in *S. pneumoniae* is concerning, as β-lactam drugs, namely penicillin G or amoxicillin, are the primary treatment for pneumococcal disease [42]. We also observed high resistance rates to ampicillin and amoxicillin in *S. aureus*, suggesting cross-resistance to other synthetic penicillins. The methicillin resistance gene (MecA) confers resistance to most beta-lactam antibiotics [43,44], potentially driving resistance to penicillin and its derivative observed in *S. aureus.*

Co-trimoxazole resistance is another pressing concern, with a high pooled resistance among *S. pneumoniae* (infection and colonization) and *E. coli* (infection). This exceeds reported rates in other populations [45]. Contrary to expectations, SCD patients showed higher co-trimoxazole-resistant *S. pneumoniae* and *E. coli* prevalence than people living with HIV, who typically receive co-trimoxazole prophylaxis [42]. While antibiotic prophylaxis may contribute to the emergence of resistance, frequent use of antibiotics for treatment primarily drives the rapid development of resistance. SCD patients may likely carry or be infected with co-trimoxazole-resistant pathogens due to prior exposure to co-trimoxazole treatment [46]. Notably, while the sickle cell trait has been shown to offer some protection against severe forms of malaria [47,48], SCD patients are still at risk and may receive antimalarial treatments, including sulfadoxine-pyrimethamine, which is commonly used in Africa [49]. This prior exposure to sulfonamides may contribute to the development of co-trimoxazole resistance. The emergence of these resistant strains can lead to treatment failures, especially in severe infections where co-trimoxazole is used as a first-line therapy.

Methicillin-resistant *Staphylococcus aureus* (MRSA) is among the six leading pathogens associated with AMR mortalities, and was responsible for more than 100,000 deaths estimated globally in 2019 [4]. Our study found a notable association between SCD and MRSA, with a pooled prevalence of MRSA colonization at 10.84%. The prevalence of MRSA carriage among SCD patients varies geographically and is influenced by demographical and local epidemiological factors. For instance, a study conducted in Ghana found a high rate of *S. aureus* carriage, but a comparatively low prevalence of MRSA (1%) among adults with SCD [20]. Similarly in Ghana, another study among children with SCD reported a nasal carriage prevalence of MRSA at 3.33% [16]. A study in Tanzania also found the rate of *S. aureus* carriage to be 42% [29] higher than that reported in Ghana. We found only one study describing MRSA infection among SCD patients with a prevalence of 63.83% higher than the 3% prevalence of MRSA lower respiratory tract infections (LRTI) in SCD adult patients with severe acute chest syndrome (ACS), reported in a French study in 2021 [50]. The emergence of MRSA among SCD patients is a crucial area of study, given the unique vulnerabilities of this population. While the prevalence of MRSA reported may be low, the potential for severe outcomes from infections necessitates ongoing surveillance and research. Further studies into local epidemiological patterns of MRSA carriage or infection will be essential for developing effective prevention and treatment protocols tailored to this at-risk group.

*Escherichia coli*, a facultative Gram-negative bacterium, is a common cause of urinary tract infection (UTI) worldwide [51]. Our study identified uropathogenic *E. coli* (UPEC) isolated from urinary tract infection exhibiting notable resistance to cephalosporins and sulfonamides. The rates of resistance to cephalosporins ranged from 24.89 to 30.43%, similar to those reported by Bunduki et al. [52]. This concerning level of resistance may be attributed to the production of extended-spectrum beta-lactamase (ESBL) in the *E. coli* isolates. The spread of these strains could compromise treatment options, necessitating the use of carbapenems as the best option for treating ESBL-producing uropathogenic *E. coli* [53,54]. Carbapenems are preferably used for treating UTIs caused by extensively drug-resistant isolates with no or limited treatment options [52]. However, relying on carbapenems as routine first-line treatment may accelerate the development of resistance, ultimately limiting the effectiveness of these antibiotics as a last resort.

This study had some potential limitations. Firstly, the studies included in this systematic review were clustered in just seven countries, leaving significant knowledge gaps in regions with high SCD prevalence, and hence, the AMR prevalence and OR reported may be an underestimation. Secondly, most of the studies lacked control participants of non-SCD patients, making it difficult to estimate the risk of AMR infection or carriage attributable to SCD.

## 5. Conclusions

This systematic review reveals alarming rates of AMR in SCD patients, spanning multiple bacterial pathogens and multiple antimicrobial drugs. Notably, SCD patients have an increased risk of resistance to penicillin and co-trimoxazole across various bacterial pathogens, including *S. pneumoniae*, *S. aureus*, and *E. coli*. The high prevalence of resistance, especially to commonly used antibiotics such as β-lactams and sulfonamides, underscores the urgent need for effective monitoring of antimicrobial use in SCD care worldwide. To better grasp the global impact of AMR on sickle cell disease, more research is crucial, especially in regions with high SCD burden. This will help fill knowledge gaps, accurately quantify the worldwide AMR burden on SCD patients, and inform evidence-based interventions.

## Figures and Tables

**Table 1:** Characteristics of included studies.

Authors, Year ^(Ref)^	Country	Study Design	Year of Study	Study Population	Age Category	No. SCD Patients	Isolate Type	Isolate Site	Organism(s)	AST Method
Abdulmanea et al., 2023 [15]	Saudi Arabia	Prospective	2017–2021	Sickle cell disease and non-Sickle cell disease patients	All ages	47	Infection	Blood	*S. aureus*	VITEK 2
Donkor et al., 2013 [19]	Ghana	Prospective	2006–2007	HbSS+ and HbSS- children	Children	142	Carriage	Nasal and Nasopharynx	*S. aureus*, *S. pneumoniae*	Disc diffusion
Dayie et al., 2022 [20]	Ghana	Prospective	2016–2017	Sickle cell disease adults	Adults	200	Carriage	Nasopharynx	*S. aureus*	Disc diffusion
Dayie et al., 2021 [21]	Ghana	Prospective	2016–2017	Sickle cell disease children	Children	202	Carriage	Nasopharynx	*S. aureus*	Disc diffusion
Mava et al., 2012 [22]	Nigeria	Not reported	2005–2008	HbSS+ and HbSS- children	Children	250	Infection	Urinary tract	*E. coli*, *Proteus spp, Coliforms*, *Klebsiella spp, S. aureus*, *Salmonella spp*	Disc diffusion
Lo et al., 2023 [23]	Nigeria	Not reported	2014–2018	HbSS+ and HbSS- children	Children	192	Infection	Blood and CSF	*S. pneumoniae*	Broth microdilution
Said et al., 2022 [24]	Tanzania	Prospective	2021	HIV, Diabetes Mellitus, and Sickle cell disease children	Children	404	Carriage	Rectum	*Enterobacteriaceae*	Disc diffusion
Dibbasey et al., 2023 [14]	Gambia	Retrospective	2015–2022	Sickle cell disease patients	All ages	159	Infection	Blood	*S. pneumoniae*, *S. aureus*	Disc diffusion
Steele et al., 1996 [25]	USA	Not reported	1994–1995	Sickle cell disease and non-Sickle cell disease patients	Children	596	Carriage	Nasopharynx	*S. pneumoniae*	Disc diffusion
Dayie et al., 2018 [26]	Ghana	Prospective	2016–2017	Sickle cell disease patients	All ages	402	Carriage	Nasopharynx	*S. pneumoniae*	Disc diffusion
Norris et al., 2003 [27]	USA	Prospective	1993–2001	Sickle cell disease children	Children	105	Infection	Blood	*S. pneumoniae*	E-test
Miller et al., 2005 [28]	USA	Retrospective	1994–1995	Sickle cell disease patients	Not reported	42	Carriage	Nasopharynx	*S. pneumoniae*	E-test
Appiah et al., 2020 [16]	Ghana	Prospective	2018	Sickle cell disease and non-Sickle cell disease patients	Children	220	Carriage	Nasal	*S. aureus*	Disc diffusion
Mutagonda et al., 2022 [29]	Tanzania	Prospective	2021	Sickle cell disease children	Children	204	Carriage	Nasopharynx	*S. pneumoniae*, *S. aureus*	Disc diffusion
Subudhi et al., 2021 [30]	India	Prospective	2019–2020	Sickle cell anemia patients at the MICU	Adults	190	Infection	Urinary tract	*S. aureus, S. pneumoniae, E. coli*, *K. pneumoniae*, *P. aeruginosa*, *A. baumannii*	Disc diffusion
Brown et al., 2003 [31]	Nigeria	Prospective	1999–2000	Sickle cell disease and non-Sickle cell disease patients	Children	342	Infection	Urinary tract	*E. coli*, *K. pneumoniae*, *Salmonellae* spp., *S. aureus*	Disc diffusion
Daw et al., 1997 [32]	USA	Not reported	1994–1995	Sickle cell disease children	Children	312	Carriage	Nasopharynx	*S. pneumoniae*	E-test
Sangeda et al., 2024 [33]	Tanzania	Prospective	2015	Sickle cell disease children	Children	250	Infection	Urinary tract	*E. coli*, *Staphylococcus* spp., *Klebsiella* spp., *Proteus* spp., *Pseudomonas* spp.	Disc diffusion

USA: United States; HIV: human immunodeficiency virus; MICU: medical intensive care unit; HbSS+: hemoglobin SS-positive; and HbSS-: hemoglobin SS-negative.

**Table 2:** Pooled resistance to selected antibiotics.

Organism	Antibiotic Class	Antibiotic	No. Studies	Pooled Resistance (%) [95% CI]	Heterogeneity I^2^ (%), *p*-Value
*S. aureus* (Infection)	Cephalosporins	Cefuroxime	2	34.82 [2.51; 76.56]	61.4, *p* = 0.1074
	Fluoroquinolones	Ciprofloxacin	3	16.10 [7.03; 27.31]	0, *p* = 0.8855
	Penicillins	Penicillin	2	99.99 [94.87; 100.00]	0, *p* = 0.8184
		Ampicillin	3	98.15 [49.83; 100.00]	80.4, *p* = 0.0060
		Amoxicillin	3	77.82 [61.93; 91.16]	0, *p* = 0.8369
	Aminoglycosides	Gentamicin	3	42.48 [3.02; 87.98]	50.7, *p* = 0.1313
	Macrolides	Erythromycin	2	53.94 [15.57; 89.98]	80.4, *p* = 0.0241
	Sulfonamides	Co-trimoxazole	3	24.78 [0.00; 72.08]	81.3, *p* = 0.0011
*S. aureus* (Colonization)	Fluoroquinolones	Ciprofloxacin	3	15.57 [7.50; 25.78]	80.5, *p* = 0.0059
	Penicillins	Penicillin	5	90.47 [57.19; 100.00]	98.3, *p* < 0.0001
	Aminoglycosides	Gentamicin	4	16.89 [8.76; 26.89]	80.3, *p* = 0.0016
	Lincosamides	Clindamycin	3	11.02 [2.08; 25.13]	90.5, *p* < 0.0001
	Macrolides	Erythromycin	5	30.25 [10.92; 53.98]	95.4, *p* < 0.0001
	Tetracyclines	Tetracycline	3	30.93 [16.01; 48.17]	89.7, *p* < 0.0001
	Sulfonamides	Co-trimoxazole	3	31.61 [14.47; 51.62]	77.6, *p* = 0.0114
*S. pneumoniae* (Infection)	Penicillins	Penicillin	3	46.61 [24.58; 69.21]	52.6, *p* = 0.1214
	Macrolides	Erythromycin	2	6.50 [0.00; 49.50]	62.7, *p* = 0.1018
	Sulfonamides	Co-trimoxazole	3	74.26 [7.14; 100.00]	95.5, *p* < 0.0001
*S. pneumoniae* (Colonization)	Penicillins	Penicillin	5	47.27 [36.13; 58.55]	66.6, *p* = 0.0175
	Macrolides	Erythromycin	3	18.75 [6.01; 35.48]	54.1, *p* = 0.1133
	Sulfonamides	Co-trimoxazole	3	84.99 [70.32; 95.75]	48.1, *p* = 0.1455
*E. coli* (Infection)	Cephalosporins	Cefuroxime	3	30.43 [11.52; 52.5]	30, *p* = 0.24
		Ceftriaxone	4	24.89 [5.94; 49.18]	66, *p* = 0.03
	Sulfonamides	Co-trimoxazole	4	93.17 [72.10; 100]	70, *p* = 0.02

**Table 3:** The pooled odds ratio for antimicrobial resistance in *Staphylococcus aureus* isolated from SCD patients compared to non-SCD patients (HbAA).

*S. aureus*	Antibiotic Class	Antibiotic	No. Studies	OR [95% CI]	Heterogeneity I^2^ (%), *p*-Value
Infection	Penicillins	Penicillin	1	0.94 [0.04; 24.22]	Not applicable
		Ampicillin	3	0.20 [0.02; 2.39]	Not applicable
		Amoxicillin	3	1.47 [0.04; 52.77]	84.9, *p* = 0.0102
	Macrolides	Erythromycin	2	2.30 [0.73; 7.18]	0, *p* = 0.8686
	Sulfonamides	Co-trimoxazole	3	0.70 [0.16; 2.99]	0, *p* = 0.9647
Colonization	Penicillins	Penicillin	2	7.62 [0.37; 155.87]	Not applicable
		Ampicillin	1	7.62 [0.37; 155.87]	Not applicable
	Macrolides	Erythromycin	2	2.64 [0.87; 8.02]	0, *p* = 0.5066
	Sulfonamides	Co-trimoxazole	2	0.60 [0.12; 2.97]	56.4, *p* = 0.1301

## Data Availability

Not applicable.

## Supplementary Materials

The following supporting information can be downloaded at: https://www.mdpi.com/article/10.3390/idr17020032/s1. Figure S1: Pooled resistance of *Staphylococcus aureus* isolated from infections among SCD patients; Figure S2: Pooled resistance of *Staphylococcus aureus* colonizing SCD patients; Figure S3: Pooled resistance of *Streptococcus pneumoniae* isolated from infections among SCD patients; Figure S4: Pooled resistance of *Streptococcus pneumoniae* colonizing SCD patients; Figure S5: Pooled resistance of Escherichia coli causing urinary tract infection among SCD patients; Table S1: Search strategy; Table S2: Patient characteristics; Table S3: Quality of included studies; and PRISMA_checklist_SCDandAMR2024.

## References

[1] Giacomini E., Perrone V., Alessandrini D., Paoli D., Nappi C., Degli Esposti L. (2021). Evidence of Antibiotic Resistance from Population-Based Studies: A Narrative Review. Infect. Drug Resist..

[2] Salam M.d.A., Al-Amin M.d.Y., Salam M.T., Pawar J.S., Akhter N., Rabaan A.A., Alqumber M.A.A. (2023). Antimicrobial Resistance: A Growing Serious Threat for Global Public Health. Healthcare.

[3] Donkor E.S., Odoom A., Osman A.-H., Darkwah S., Kotey F.C.N. (2024). A Systematic Review on Antimicrobial Resistance in Ghana from a One Health Perspective. Antibiotics.

[4] Murray C.J.L., Ikuta K.S., Sharara F., Swetschinski L., Robles Aguilar G., Gray A., Han C., Bisignano C., Rao P., Wool E. (2022). Global burden of bacterial antimicrobial resistance in 2019: A systematic analysis. Lancet.

[5] Founou R.C., Founou L.L., Essack S.Y. (2017). Clinical and economic impact of antibiotic resistance in developing countries: A systematic review and meta-analysis. PLoS ONE.

[6] Levy S.B., Marshall B. (2004). Antibacterial resistance worldwide: Causes, challenges and responses. Nat. Med..

[7] Booth C., Inusa B., Obaro S.K. (2010). Infection in sickle cell disease: A review. Int. J. Infect. Dis. IJID Off. Publ. Int. Soc. Infect. Dis..

[8] Elendu C., Amaechi D.C., Alakwe-Ojimba C.E., Elendu T.C., Elendu R.C., Ayabazu C.P., Aina T.O., Aborisade O., Adenikinju J.S. (2023). Understanding Sickle cell disease: Causes, symptoms, and treatment options. Medicine.

[9] Weatherall D.J. (2010). The inherited diseases of hemoglobin are an emerging global health burden. Blood.

[10] Bailey M., Gibbs M., Dani N., Mendell A., Thompson M. (2019). Burden of Illness of Sickle Cell Disease in Countries of the Middle East: A Systematic Literature Review. Blood.

[11] GBD 2021 Sickle Cell Disease Collaborators (2023). Global, regional, and national prevalence and mortality burden of sickle cell disease, 2000–2021: A systematic analysis from the Global Burden of Disease Study 2021. Lancet Haematol..

[12] Williams T.N. (2016). Sickle Cell Disease in Sub-Saharan Africa. Hematol. Oncol. Clin. N. Am..

[13] Battersby A.J., Knox-Macaulay H.H.M., Carrol E.D. (2010). Susceptibility to invasive bacterial infections in children with sickle cell disease. Pediatr. Blood Cancer.

[14] Dibbasey M., Dahaba M., Sarfo F., Jallow-Manneh I., Ceesay B., Umukoro S., Diop M.F., Amambua-Ngwa A. (2023). Laboratory indices of hospitalized sickle cell disease patients, prevalence and antimicrobial susceptibility of pathogenic bacterial isolates at MRCG ward in the Gambia. BMC Infect. Dis..

[15] Abdulmanea A.A., Alharbi N.S., Somily A.M., Khaled J.M., Algahtani F.H. (2023). The Prevalence of the Virulence Genes of *Staphylococcus aureus* in Sickle Cell Disease Patients at KSUMC, Riyadh, Saudi Arabia. Antibiotics.

[16] Appiah V.A., Pesewu G.A., Kotey F.C.N., Boakye A.N., Duodu S., Tette E.M.A., Nyarko M.Y., Donkor E.S. (2020). *Staphylococcus aureus* nasal colonization among children with sickle cell disease at the children’s hospital, accra: Prevalence, risk factors, and antibiotic resistance. Pathogens.

[17] Page M.J., Moher D., Bossuyt P.M., Boutron I., Hoffmann T.C., Mulrow C.D., Shamseer L., Tetzlaff J.M., Akl E.A., Brennan S.E. (2021). PRISMA 2020 explanation and elaboration: Updated guidance and exemplars for reporting systematic reviews. BMJ.

[18] Ouzzani M., Hammady H., Fedorowicz Z., Elmagarmid A. (2016). Rayyan-a web and mobile app for systematic reviews. Syst. Rev..

[19] Donkor E.S., Foster-Nyarko E., Enweronu-Laryea C.C. (2013). Relationship between antibiotic resistance and sickle cell anemia: Preliminary evidence from a pediatric carriage study in Ghana. Infect. Drug Resist..

[20] Dayie N.T., Sekoh D.N., Tetteh-Quarcoo P.B., Dayie A.D., Osei M.-M., Kotey F.C., Donkor E.S. (2022). *Staphylococcus aureus* Nasopharyngeal Carriage and Antimicrobial Resistance among Adults with Sickle Cell Disease at the Korle Bu Teaching Hospital in Accra, Ghana. Microbiol. Insights.

[21] Dayie N.T.K.D., Sekoh D.N.K., Kotey F.C.N., Egyir B., Tetteh-Quarcoo P.B., Adutwum-Ofosu K.K., Ahenkorah J., Osei M.-M., Donkor E.S. (2021). Nasopharyngeal Carriage of Methicillin-Resistant *Staphylococcus aureus* (MRSA) among Sickle Cell Disease (SCD) Children in the Pneumococcal Conjugate Vaccine Era. Infect. Dis. Rep..

[22] Mava Y., Bello M., Ambe J.P., Zailani S.B. (2012). Antimicrobial sensitivity pattern of organisms causing urinary tract infection in children with sickle cell anemia in Maiduguri, Nigeria. Niger. J. Clin. Pract..

[23] Lo S.W., Hawkins P.A., Jibir B., Hassan-Hanga F., Gambo M., Olaosebikan R., Olanipekun G., Munir H., Kocmich N., Rezac-Elgohary A. (2023). Molecular characterization of *streptococcus pneumoniae* causing disease among children in nigeria during the introduction of pcv10 (Gsk). Microb. Genomics.

[24] Said M.M., Msanga D.R., Mtemisika C.I., Silago V., Mirambo M.M., Mshana S.E. (2022). Extended Spectrum β-Lactamase Producing Lactose Fermenting Bacteria Colonizing Children with Human Immunodeficiency Virus, Sickle Cell Disease and Diabetes Mellitus in Mwanza City, Tanzania: A Cross-Sectional Study. Trop. Med. Infect. Dis..

[25] Steele R.W., Warrier R., Unkel P.J., Foch B.J., Howes R.F., Shah S., Williams K., Moore S., Jue S.J. (1996). Colonization with antibiotic-resistant *Streptococcus pneumoniae* in children with sickle cell disease. J. Pediatr..

[26] Dayie N.T.K.D., Tetteh-Ocloo G., Labi A.-K., Olayemi E., Slotved H.-C., Lartey M., Donkor E.S. (2018). Pneumococcal carriage among sickle cell disease patients in Accra, Ghana: Risk factors, serotypes and antibiotic resistance. PLoS ONE.

[27] Norris C.F., Smith-Whitley K., McGowan K.L. (2003). Positive blood cultures in sickle cell disease: Time to positivity and clinical outcome. J. Pediatr. Hematol. Oncol..

[28] Miller M.L., Obert C.A., Gao G., Daw N.C., Flynn P., Tuomanen E. (2005). Cephalosporin-resistant Pneumococci and sickle cell disease. Emerg. Infect. Dis..

[29] Mutagonda R.F., Bwire G., Sangeda R.Z., Kilonzi M., Mlyuka H., Ndunguru J., Jonathan A., Makani J., Minja I.K., Ruggajo P. (2022). Nasopharyngeal Carriage and Antibiogram of Pneumococcal and Other Bacterial Pathogens from Children with Sickle Cell Disease in Tanzania. Infect. Drug Resist..

[30] Subudhi M., Jagatheeswary P.A.T., Sahu S.K., Das S.K., Subudhi K.B., Rout R.R. (2021). Incidence and variation of microbiological profile of catheter-associated urinary tract infection in precise comorbidities associated with tribal sickle cell anemic patients of medical intensive care unit in a tribal tertiary care center. J. Appl. Hematol..

[31] Brown B.J., Asinobi A.O., Fatunde O.J., Osinusi K., Fasina N.A. (2003). Antimicrobial sensitivity pattern of organisms causing urinary tract infection in children with sickle cell anaemia in Ibadan, Nigeria. West Afr. J. Med..

[32] Daw N.C., Wilimas J.A., Wang W.C., Presbury G.J., Joyner R.E., Harris S.C., Davis Y., Chen G., Joan Chesney P. (1997). Nasopharyngeal carriage of penicillin-resistant *Streptococcus pneumoniae* in children with sickle cell disease. Pediatrics.

[33] Sangeda R., Yohana J., Jonathan A., Manyanga V., Soka D., Makani J. (2024). Prevalence of Urinary Tract Infections and Antibiogram of Bacteria Isolated From Children With Sickle Cell Disease in Tanzania. CUREUS J. Med. Sci..

[34] Gajic I., Kabic J., Kekic D., Jovicevic M., Milenkovic M., Mitic Culafic D., Trudic A., Ranin L., Opavski N. (2022). Antimicrobial Susceptibility Testing: A Comprehensive Review of Currently Used Methods. Antibiotics.

[35] Webber D.M., Wallace M.A., Burnham C.-A.D. (2022). Stop Waiting for Tomorrow: Disk Diffusion Performed on Early Growth Is an Accurate Method for Antimicrobial Susceptibility Testing with Reduced Turnaround Time. J. Clin. Microbiol..

[36] Li K., Zhong W., Li P., Ren J., Jiang K., Wu W. (2023). Antibacterial mechanism of lignin and lignin-based antimicrobial materials in different fields. Int. J. Biol. Macromol..

[37] Gupta P., Khare V., Kumar D., Ahmad A., Banerjee G., Singh M. (2015). Comparative evaluation of disc diffusion and E-test with broth micro-dilution in Susceptibility testing of amphotericin B, voriconazole and caspofungin against clinical Aspergillus isolates. J. Clin. Diagn. Res..

[38] Pincus D.H. (2006). Microbial Identification Using the Biomérieux VITEK^®^ 2 System. Encyclopedia of Rapid Microbiological Methods.

[39] Salam M.d.A., Al-Amin M.d.Y., Pawar J.S., Akhter N., Lucy I.B. (2023). Conventional methods and future trends in antimicrobial susceptibility testing. Saudi J. Biol. Sci..

[40] Cober M.P., Phelps S.J. (2010). Penicillin Prophylaxis in Children with Sickle Cell Disease. J. Pediatr. Pharmacol. Ther. JPPT.

[41] van Aalst M., Lötsch F., Spijker R., van der Meer J.T.M., Langendam M.W., Goorhuis A., Grobusch M.P., de Bree G.J. (2018). Incidence of invasive pneumococcal disease in immunocompromised patients: A systematic review and meta-analysis. Travel Med. Infect. Dis..

[42] Olaru I.D., Tacconelli E., Yeung S., Ferrand R.A., Stabler R.A., Hopkins H., Aiken A.M., Kranzer K. (2021). The association between antimicrobial resistance and HIV infection: A systematic review and meta-analysis. Clin. Microbiol. Infect..

[43] Mlynarczyk-Bonikowska B., Kowalewski C., Krolak-Ulinska A., Marusza W. (2022). Molecular Mechanisms of Drug Resistance in *Staphylococcus aureus*. Int. J. Mol. Sci..

[44] Peacock S.J., Paterson G.K. (2015). Mechanisms of Methicillin Resistance in *Staphylococcus aureus*. Annu. Rev. Biochem..

[45] Zachariah R., Harries A.D., Spielmann M.P., Arendt V., Nchingula D., Mwenda R., Courtielle O., Kirpach P., Mwale B., Salaniponi F.M.L. (2002). Changes in Escherichia coli resistance to co-trimoxazole in tuberculosis patients and in relation to co-trimoxazole prophylaxis in Thyolo, Malawi. Malawi Med. J..

[46] Mwenya D.M., Charalambous B.M., Phillips P.P.J., Mwansa J.C.L., Batt S.L., Nunn A.J., Walker S., Gibb D.M., Gillespie S.H. (2010). Impact of cotrimoxazole on carriage and antibiotic resistance of *Streptococcus pneumoniae* and Haemophilus influenzae in HIV-infected children in Zambia. Antimicrob. Agents Chemother..

[47] Ladu A.I., Kadaura M.U., Dauda M., Baba A.S., Zango N.G., Jeffery C., Farate A., Adekile A., Bates I. (2024). Malaria Infection in Patients with Sickle Cell Disease in Nigeria: Association with Markers of Hyposplenism. Hemoglobin.

[48] Mwaiswelo R.O., Mawala W., Iversen P.O., de Montalembert M., Luzzatto L., Makani J. (2020). Sickle cell disease and malaria: Decreased exposure and asplenia can modulate the risk from Plasmodium falciparum. Malar. J..

[49] Feikin D.R., Dowell S.F., Nwanyanwu O.C., Klugman K.P., Kazembe P.N., Barat L.M., Graf C., Bloland P.B., Ziba C., Huebner R.E. (2000). Increased carriage of trimethoprim/sulfamethoxazole-resistant *Streptococcus pneumoniae* in Malawian children after treatment for malaria with sulfadoxine/pyrimethamine. J. Infect. Dis..

[50] Elabbadi A., Voiriot G., Tristan A., Gibelin A., Verdet C., Djibré M., Santin A., Jutant E.-M., Lopinto J., Vandenesch F. (2021). Lower respiratory tract infection with *Staphylococcus aureus* in sickle-cell adult patients with severe acute chest syndrome—The STAPHACS Study. Haematologica.

[51] Raeispour M., Ranjbar R. (2018). Antibiotic resistance, virulence factors and genotyping of Uropathogenic Escherichia coli strains. Antimicrob. Resist. Infect. Control.

[52] Bunduki G.K., Heinz E., Phiri V.S., Noah P., Feasey N., Musaya J. (2021). Virulence factors and antimicrobial resistance of uropathogenic Escherichia coli (UPEC) isolated from urinary tract infections: A systematic review and meta-analysis. BMC Infect. Dis..

[53] Pootong A., Mungkornkeaw N., Norrapong B., Cowawintaweewat S. (2018). Phylogenetic background, drug susceptibility and virulence factors of uropathogenic E. coli isolate in a tertiary university hospital in central Thailand. Trop. Biomed..

[54] Terlizzi M.E., Gribaudo G., Maffei M.E. (2017). UroPathogenic Escherichia coli (UPEC) Infections: Virulence Factors, Bladder Responses, Antibiotic, and Non-antibiotic Antimicrobial Strategies. Front. Microbiol..

