# Supplementary material for: Sickle Cell Disease and Antimicrobial Resistance: A Systematic Review and Meta-Analysis
Source: Infect Dis Rep. 2025 Apr 14;17(2):32. doi: 10.3390/idr17020032 (PMC12026643; doi:10.3390/idr17020032)
Supplement: Supplementary file 1 [file idr-17-00032-s001.zip › Figures_supplementary_material.pdf]

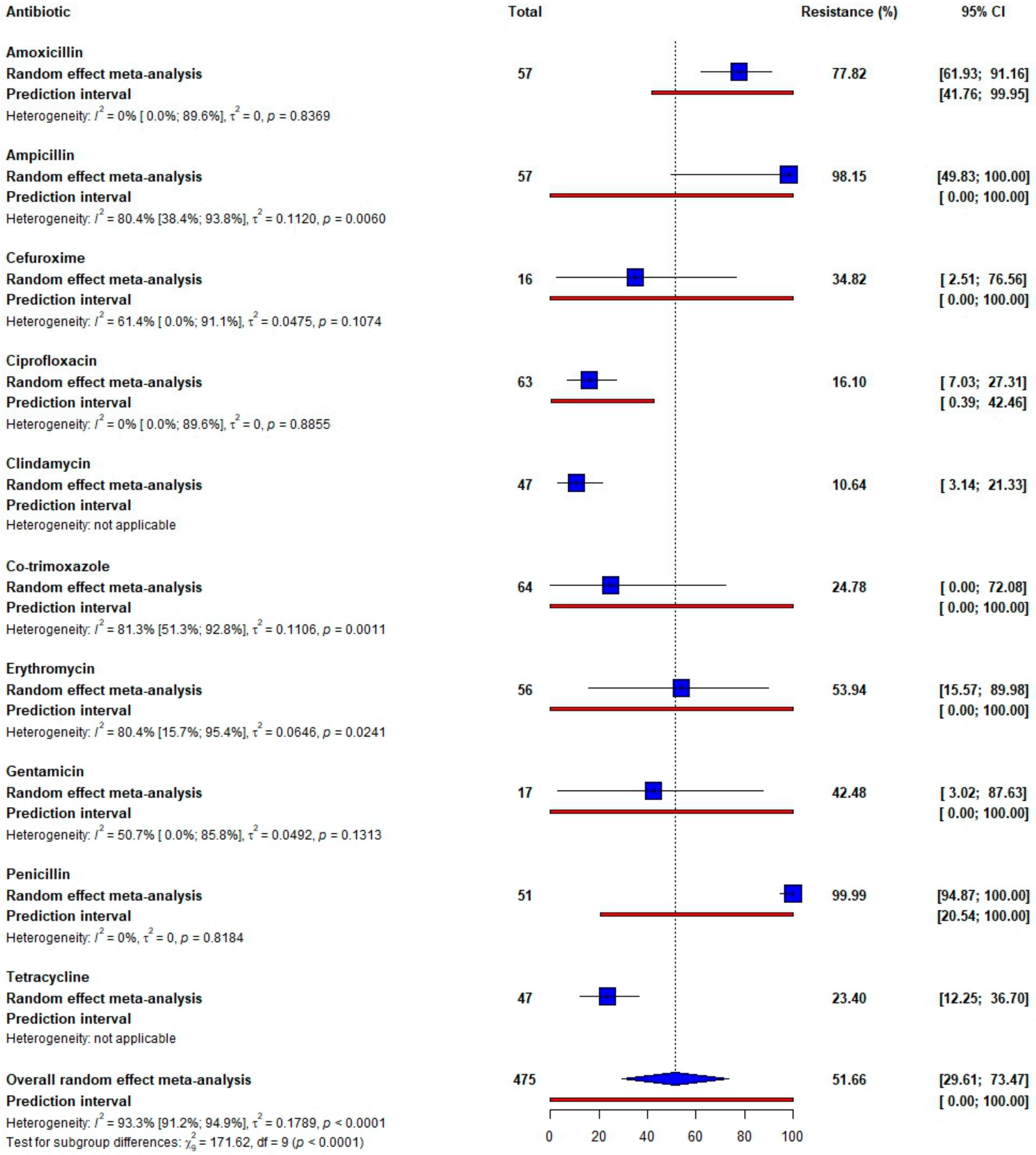

Figure S1. Pooled resistance of *Staphylococcus aureus* isolated from infections among SCD patients.

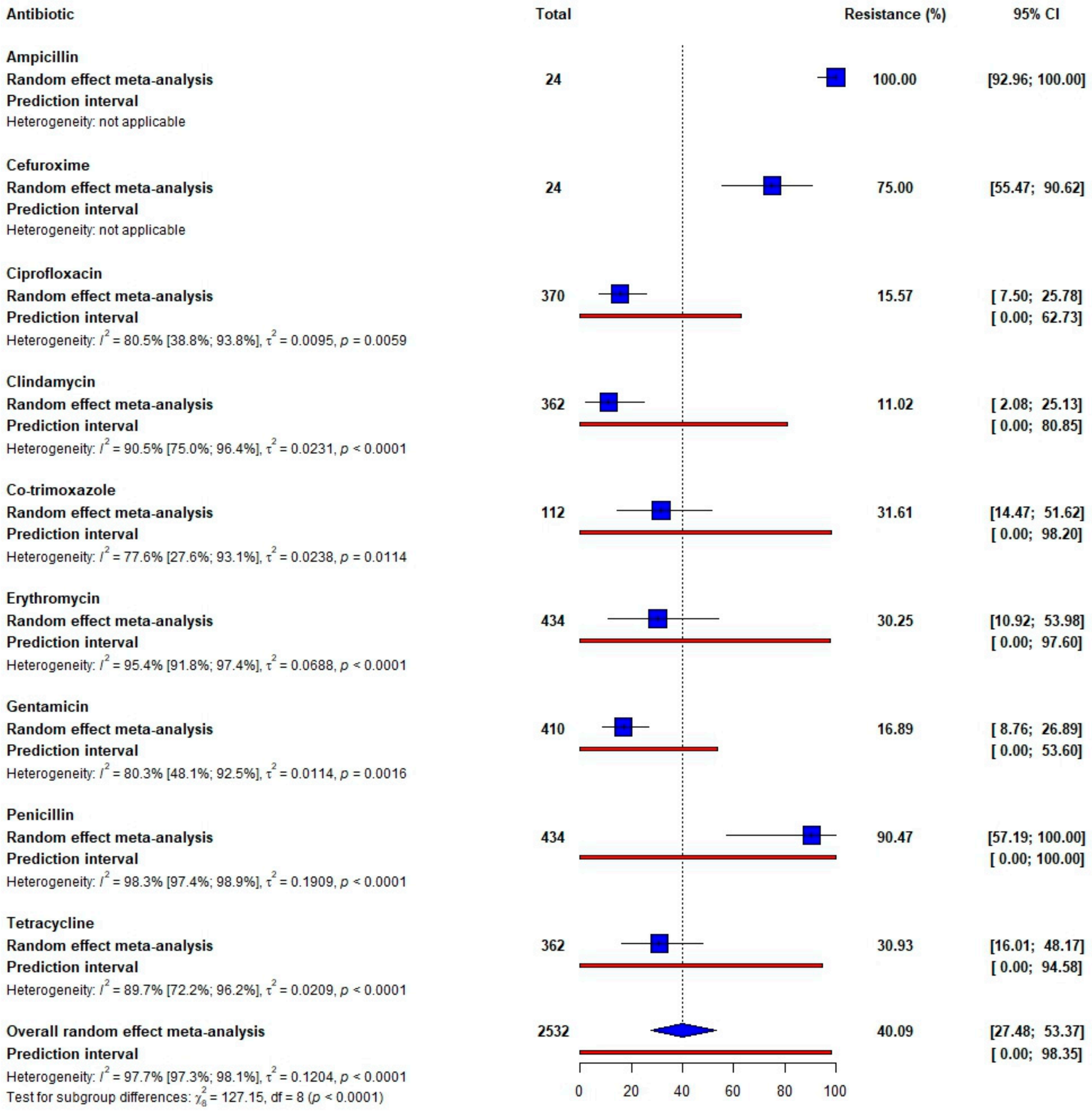

Figure S2. Pooled resistance of *Staphylococcus aureus* colonizing SCD patients.

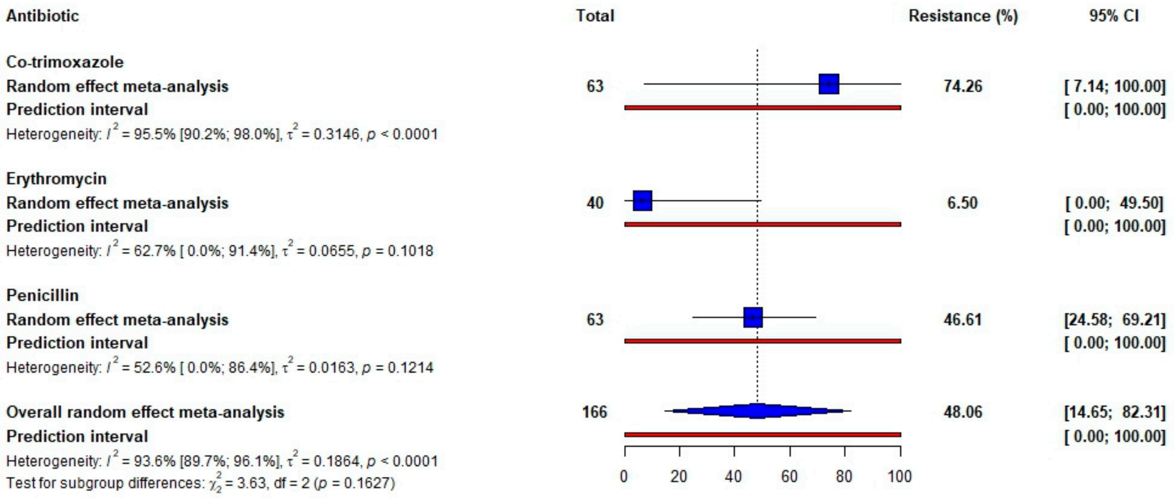

Figure S3. Pooled resistance of *Streptococcus pneumoniae* isolated from infections among SCD patients

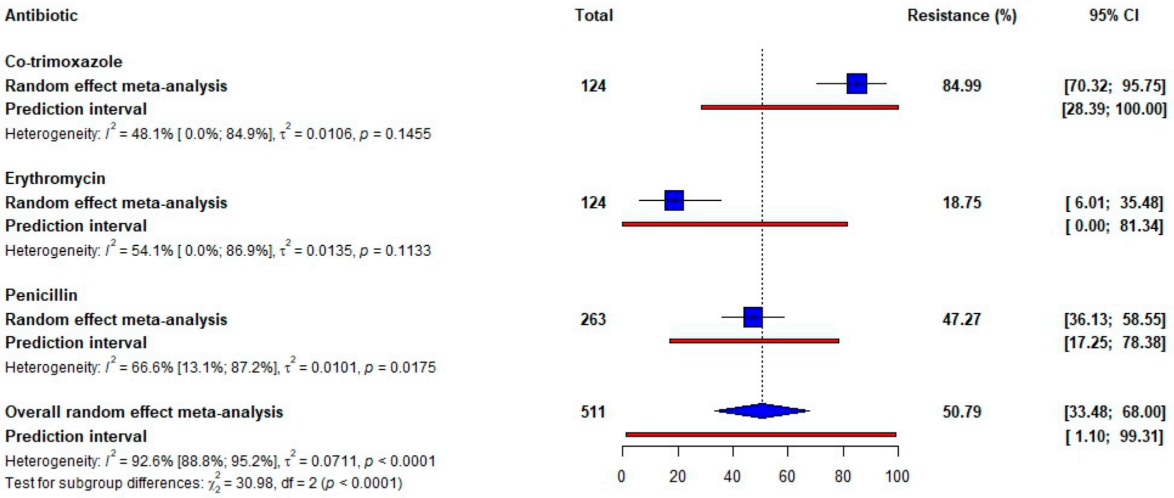

Figure S4. Pooled resistance of *Streptococcus pneumoniae* colonizing SCD patients

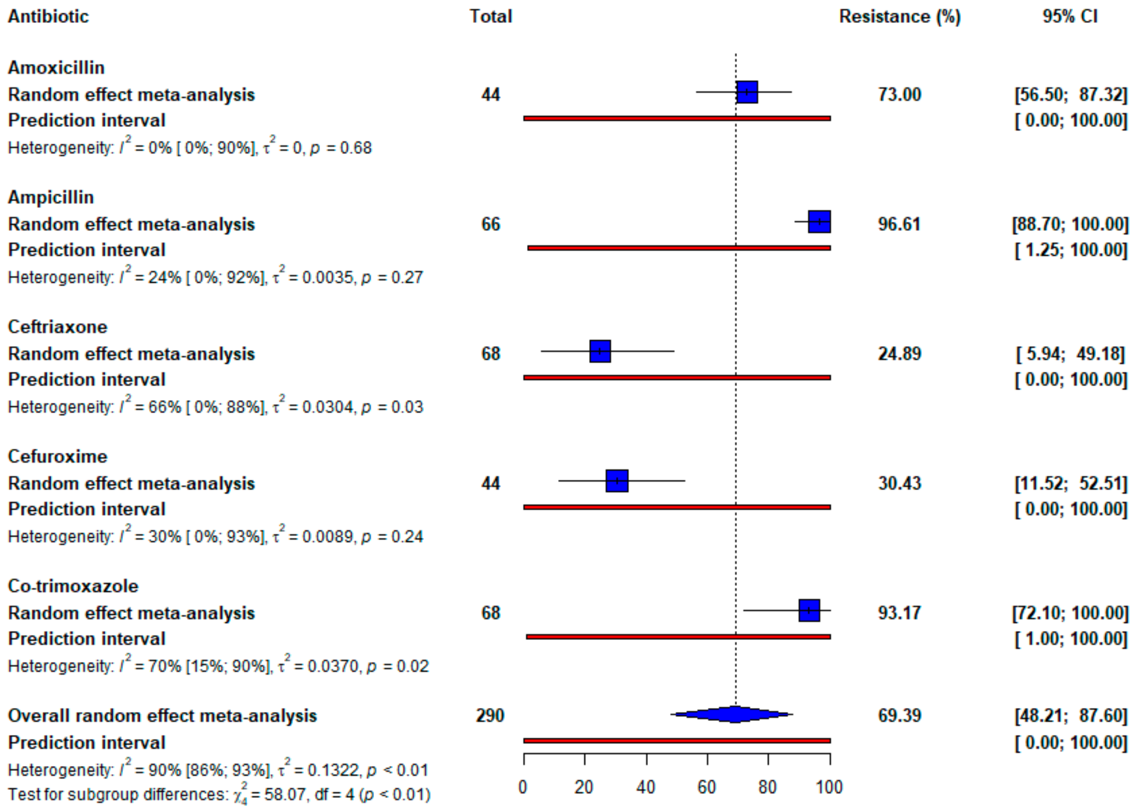

Figure S5. Pooled resistance of *Escherichia coli* causing urinary tract infection among SCD patients
