# Supplementary material for: Sickle Cell Disease and Antimicrobial Resistance: A Systematic Review and Meta-Analysis
Source: Infect Dis Rep. 2025 Apr 14;17(2):32. doi: 10.3390/idr17020032 (PMC12026643; doi:10.3390/idr17020032)
Supplement: Supplementary file 1 [file idr-17-00032-s001.zip › Table_S1_supplementary_material.pdf]

Table S1. Detailed search strategy

Database	Keywords	Filters
**PubMed**	(“Antimicrobial resistance” OR “Antibiotic resistance” OR “AMR”) AND (“Staphylococcus” OR “Enterococcus” OR “Streptococcus” OR “Escherichia coli” OR “Klebsiella” OR “Pseudomonas” OR “Acinetobacter” OR “Enterobacter” OR “Salmonellae” OR “Shigella” OR “Enterobacteriaceae” OR “Enterobacteria”) AND (“Sickle cell anemia” OR “Sickle cell anaemia” OR “Sickle cell disease” OR “Sickle cell disorder” OR “SCD” OR “Hemoglobin SS disease” OR “HbSS” OR “Sickle cell hemoglobinopathy” OR “Hemoglobin SC disease” OR “HbSC” OR “Sickle cell beta thalassemia”)	English
**Scopus**	(“Antimicrobial resistance” OR “Antibiotic resistance” OR “AMR”) AND (“Staphylococcus” OR “Enterococcus” OR “Streptococcus” OR “Escherichia coli” OR “Klebsiella” OR “Pseudomonas” OR “Acinetobacter” OR “Enterobacter” OR “Salmonellae” OR “Shigella” OR “Enterobacteriaceae” OR “Enterobacteria”) AND (“Sickle cell anemia” OR “Sickle cell anaemia” OR “Sickle cell disease” OR “Sickle cell disorder” OR “SCD” OR “Hemoglobin SS disease” OR “HbSS” OR “Sickle cell hemoglobinopathy” OR “Hemoglobin SC disease” OR “HbSC” OR “Sickle cell beta thalassemia”)	Articles, English
**ScienceDirect**	(“Antimicrobial resistance” OR “Antibiotic resistance” OR “AMR”) AND (Bacteria) AND (“Sickle cell anemia” OR “Sickle cell anaemia” OR “Sickle cell disease” OR “Sickle cell disorder” OR “Sickle cell beta thalassemia”)	Research articles, English
**Web of Science**	(“Antimicrobial resistance” OR “Antibiotic resistance” OR “AMR”) AND (“Staphylococcus” OR “Enterococcus” OR “Streptococcus” OR “Escherichia coli” OR “Klebsiella” OR “Pseudomonas” OR “Acinetobacter” OR “Enterobacter” OR “Salmonellae” OR “Shigella” OR “Enterobacteriaceae” OR “Enterobacteria”) AND (“Sickle cell anemia” OR “Sickle cell anaemia” OR “Sickle cell disease” OR “Sickle cell disorder” OR “SCD” OR “Hemoglobin SS disease” OR “HbSS” OR “Sickle cell hemoglobinopathy” OR “Hemoglobin SC disease” OR “HbSC” OR “Sickle cell beta thalassemia”)	Articles, English
