# Supplementary material for: Sickle Cell Disease and Antimicrobial Resistance: A Systematic Review and Meta-Analysis
Source: Infect Dis Rep. 2025 Apr 14;17(2):32. doi: 10.3390/idr17020032 (PMC12026643; doi:10.3390/idr17020032)
Supplement: Supplementary file 1 [file idr-17-00032-s001.zip › Table_S2_supplementary_material.pdf]

Table S2. Patient characteristics

Authors, Year (Ref)	Country	Case group	Sickle genotype	Diagnosis of sickling status	Control group	Sample type	Respiratory Symptoms	Fever	Asthma	Pneumonia	Ear infection	Penicillin prophylaxis
Abdulmanea et al., 2023 [1]	Saudi Arabia	47	HbSS only	Not reported	16	Blood	None reported	None reported	None reported	None reported	None reported	No
Donkor et al., 2013 [2]	Ghana	50	HbSS only	Hemoglobin electropherosis	50	Nasopharyngeal	None reported	None reported	None reported	None reported	None reported	No
Dayie et al., 2022 [3]	Ghana	200	Specific type not stated	Not reported	None recruited	Nasopharyngeal	Yes	None reported	Yes	Yes	Yes	No
Dayie et al., 2021 [4]	Ghana	202	Specific type not stated	Not reported	None recruited	Nasopharyngeal	Yes	None reported	Yes	Yes	Yes	No
Mava et al., 2012 [5]	Nigeria	250	HbSS only	Not reported	250	Urine	None reported	Yes	None reported	None reported	None reported	No
Lo et al., 2023 [6]	Nigeria	37	Specific type not stated	Not reported	155	Blood/CSF	None reported	None reported	None reported	Yes	None reported	No
Said et al., 2022 [7]	Tanzania	126	Specific type not stated	Not reported	None recruited	Rectal/Stool	None reported	Yes	None reported	None reported	None reported	Yes
Dibbasey et al., 2023 [8]	Gambia	159	HbSS and HbSC	Hemoglobin electropherosis	None recruited	Blood	None reported	None reported	None reported	None reported	None reported	Yes
Steele et al., 1996 [9]	USA	351	Specific type not stated	Not reported	245	Nasopharyngeal	None reported	None reported	None reported	None reported	None reported	No
Dayie et al., 2018 [10]	Ghana	402	Specific type not stated	Hemoglobin electropherosis	None recruited	Nasopharyngeal	Yes	None reported	Yes	Yes	None reported	Yes

Norris et al., 2003 [11]	USA	105	Specific type not stated	Not reported	None recruited	Blood	None reported	None reported	None reported	None reported	None reported	No
Miller et al., 2005 [12]	USA	42	HbSS, HbSC and HbSB-thalassemia	Not reported	None recruited	Nasopharyngeal	None reported	None reported	None reported	None reported	None reported	No
Appiah et al., 2020 [13]	Ghana	120	HbSS and HbSC	Not reported	100	Nasal	None reported	None reported	None reported	Yes	None reported	Yes
Mutagonda et al., 2022 [14]	Tanzania	204	Specific type not stated	Not reported	None recruited	Nasopharyngeal	Yes	Yes	None reported	None reported	None reported	Yes
Subudhi et al., 2021 [15]	India	192	Specific type not stated	Not reported	None recruited	Urine	None reported	None reported	None reported	None reported	None reported	No
Brown et al., 2003 [16]	Nigeria	171	HbSS only	Not reported	171	Urine	None reported	Yes	None reported	None reported	None reported	No
Daw et al., 1997 [17]	USA	312	HbSS, HbSC and HbSB-thalassemia	Not reported	None recruited	Nasopharyngeal	None reported	None reported	None reported	None reported	None reported	Yes
Sangeda et al., 2024 [18]	Tanzania	250	Specific type not stated	Not reported	None recruited	Urine	None reported	Yes	None reported	None reported	None reported	Yes

References

1. Abdulmanea, A.A.; Alharbi, N.S.; Somily, A.M.; Khaled, J.M.; Algahtani, F.H. The Prevalence of the Virulence Genes of Staphylococcus aureus in Sickle Cell Disease Patients at KSUMC, Riyadh, Saudi Arabia. *Antibiotics* **2023**, *12*, 1221. https://doi.org/10.3390/antibiotics12071221.

2. Donkor, E.S.; Foster-Nyarko, E.; Enweronu-Laryea, C.C. Relationship between antibiotic resistance and sickle cell anemia: Preliminary evidence from a pediatric carriage study in Ghana. *Infect. Drug Resist.* **2013**, *6*, 71–77. https://doi.org/10.2147/IDR.S40062.

3. Dayie, N.T.; Sekoh, D.N.; Tetteh-Quarcoo, P.B.; Dayie, A.D.; Osei, M.-M.; Kotey, F.C.; Donkor, E.S. Staphylococcus aureus Nasopharyngeal Carriage and Antimicrobial Resistance among Adults with Sickle Cell Disease at the Korle Bu Teaching Hospital in Accra, Ghana. *Microbiol. Insights* **2022**, *15*, 11786361221133959. https://doi.org/10.1177/11786361221133959.

4. Dayie, N.T.K.D.; Sekoh, D.N.K.; Kotey, F.C.N.; Egyir, B.; Tetteh-Quarcoo, P.B.; Adutwum-Ofosu, K.K.; Ahenkorah, J.; Osei, M.-M.; Donkor, E.S. Nasopharyngeal Carriage of Methicillin-Resistant Staphylococcus aureus (MRSA) among Sickle Cell Disease (SCD) Children in the Pneumococcal Conjugate Vaccine Era. *Infect. Dis. Rep.* **2021**, *13*, 191–204. https://doi.org/10.3390/idr13010022.

5. Mava, Y.; Bello, M.; Ambe, J.P.; Zailani, S.B. Antimicrobial sensitivity pattern of organisms causing urinary tract infection in children with sickle cell anemia in Maiduguri, Nigeria. *Niger. J. Clin. Pract.* **2012**, *15*, 420–423. https://doi.org/10.4103/1119-3077.104515.

6. Lo, S.W.; Hawkins, P.A.; Jibir, B.; Hassan-Hanga, F.; Gambo, M.; Olaosebikan, R.; Olanipekun, G.; Munir, H.; Kocmich, N.; Rezac-Elgohary, A.; et al. Molecular characterization of streptococcus pneumoniae causing disease among children in nigeria during the introduction of pcv10 (Gsk). *Microb. Genomics* **2023**, *9*, 001094. https://doi.org/10.1099/mgen.0.001094.

7. Said, M.M.; Msanga, D.R.; Mtemisika, C.I.; Silago, V.; Mirambo, M.M.; Mshana, S.E. Extended Spectrum β-Lactamase Producing Lactose Fermenting Bacteria Colonizing Children with Human Immunodeficiency Virus, Sickle Cell Disease and Diabetes Mellitus in Mwanza City, Tanzania: A Cross-Sectional Study. *Trop. Med. Infect. Dis.* **2022**, *7*, 144. https://doi.org/10.3390/tropicalmed7080144.

8. Dibbasey, M.; Dahaba, M.; Sarfo, F.; Jallow-Manneh, I.; Ceesay, B.; Umukoro, S.; Diop, M.F.; Amambua-Ngwa, A. Laboratory indices of hospitalized sickle cell disease patients, prevalence and antimicrobial susceptibility of pathogenic bacterial isolates at MRCG ward in the Gambia. *BMC Infect. Dis.* **2023**, *23*, 546. https://doi.org/10.1186/s12879-023-08542-z.

9. Steele, R.W.; Warrier, R.; Unkel, P.J.; Foch, B.J.; Howes, R.F.; Shah, S.; Williams, K.; Moore, S.; Jue, S.J. Colonization with antibiotic-resistant Streptococcus pneumoniae in children with sickle cell disease. *J. Pediatr.* **1996**, *128*, 531–535. https://doi.org/10.1016/S0022-3476(96)70365-7.

10. Dayie, N.T.K.D.; Tetteh-Ocloo, G.; Labi, A.-K.; Olayemi, E.; Slotved, H.-C.; Lartey, M.; Donkor, E.S. Pneumococcal carriage among sickle cell disease patients in Accra, Ghana: Risk factors, serotypes and antibiotic resistance. *PLoS ONE* **2018**, *13*, e0206728. https://doi.org/10.1371/journal.pone.0206728.

11. Norris, C.F.; Smith-Whitley, K.; McGowan, K.L. Positive blood cultures in sickle cell disease: Time to positivity and clinical outcome. *J. Pediatr. Hematol. Oncol.* **2003**, *25*, 390–395. https://doi.org/10.1097/00043426-200305000-00008.

12. Miller, M.L.; Obert, C.A.; Gao, G.; Daw, N.C.; Flynn, P.; Tuomanen, E. Cephalosporin-resistant Pneumococci and sickle cell disease. *Emerg. Infect. Dis.* **2005**, *11*, 1192–1196. https://doi.org/10.3201/eid1108.050152.

13. Appiah, V.A.; Pesewu, G.A.; Kotey, F.C.N.; Boakye, A.N.; Duodu, S.; Tette, E.M.A.; Nyarko, M.Y.; Donkor, E.S. Staphylococcus aureus nasal colonization among children with sickle cell disease at the children's hospital, accra: Prevalence, risk factors, and antibiotic resistance. *Pathogens* **2020**, *9*, 329. https://doi.org/10.3390/pathogens9050329.

14. Mutagonda, R.F.; Bwire, G.; Sangeda, R.Z.; Kilonzi, M.; Mlyuka, H.; Ndunguru, J.; Jonathan, A.; Makani, J.; Minja, I.K.; Ruggajo, P.; et al. Nasopharyngeal Carriage and Antibiogram of Pneumococcal and Other Bacterial Pathogens from Children with Sickle Cell Disease in Tanzania. *Infect. Drug Resist.* **2022**, *15*, 4407–4418. https://doi.org/10.2147/IDR.S367873.

15. Subudhi, M.; Jagatheeswary, P.A.T.; Sahu, S.K.; Das, S.K.; Subudhi, K.B.; Rout, R.R. Incidence and variation of microbiological profile of catheter-associated urinary tract infection in precise comorbidities associated with tribal sickle cell anemic patients of medical intensive care unit in a tribal tertiary care center. *J. Appl. Hematol.* **2021**, *12*, 140–146. https://doi.org/10.4103/joah.joah_184_20.

16. Brown, B.J.; Asinobi, A.O.; Fatunde, O.J.; Osinusi, K.; Fasina, N.A. Antimicrobial sensitivity pattern of organisms causing urinary tract infection in children with sickle cell anaemia in Ibadan, Nigeria. *West Afr. J. Med.* **2003**, *22*, 110–113.

17. Daw, N.C.; Wilimas, J.A.; Wang, W.C.; Presbury, G.J.; Joyner, R.E.; Harris, S.C.; Davis, Y.; Chen, G.; Joan Chesney, P. Nasopharyngeal carriage of penicillin-resistant streptococcus pneumoniae in children with sickle cell disease. *Pediatrics* **1997**, *99*, 594–595. https://doi.org/10.1542/peds.99.4.e7.

18. Sangeda, R.; Yohana, J.; Jonathan, A.; Manyanga, V.; Soka, D.; Makani, J. Prevalence of Urinary Tract Infections and Antibiogram of Bacteria Isolated From Children With Sickle Cell Disease in Tanzania. *CUREUS J. Med. Sci.* **2024**, *16*, e58786. https://doi.org/10.7759/cureus.58786.
