# Supplementary material for: Sickle Cell Disease and Antimicrobial Resistance: A Systematic Review and Meta-Analysis
Source: Infect Dis Rep. 2025 Apr 14;17(2):32. doi: 10.3390/idr17020032 (PMC12026643; doi:10.3390/idr17020032)
Supplement: Supplementary file 1 [file idr-17-00032-s001.zip › Table_S3_supplementary_material.pdf]

Table S3. Quality of included studies

Authors, Year (Ref)	Study background and rationale	Objectives	Study design	Study setting	Eligibility criteria	Definition (Variables)	Data sources	Bias	Study size	Statistical methods	Included participants	Descriptive data	Outcome data	Key results and interpretation	Limitation
Abdulmanea et al., 2023 [1]	Yes	Yes	No	Yes	No	No	Yes	No	No	Yes	Yes	Yes	No	Yes	Yes
Donkor et al., 2013 [2]	Yes	Yes	Yes	Yes	Yes	No	No	No	No	Yes	Yes	Yes	No	Yes	Yes
Dayie et al., 2022 [3]	Yes	Yes	Yes	Yes	No	No	No	No	No	Yes	Yes	Yes	No	Yes	Yes
Dayie et al., 2021 [4]	Yes	Yes	Yes	Yes	No	No	No	No	No	Yes	Yes	Yes	No	Yes	Yes
Mava et al., 2012 [5]	Yes	Yes	No	Yes	Yes	No	No	No	No	No	Yes	Yes	No	Yes	No
Lo et al., 2023 [6]	Yes	Yes	No	Yes	Yes	Yes	No	No	No	Yes	Yes	No	No	Yes	No
Said et al., 2022 [7]	Yes	Yes	Yes	Yes	Yes	No	Yes	No	No	Yes	Yes	Yes	No	Yes	No
Dibbasey et al., 2023 [8]	Yes	Yes	Yes	Yes	Yes	No	Yes	No	Yes	Yes	Yes	Yes	No	Yes	Yes
Steele et al., 1996 [9]	Yes	Yes	No	Yes	Yes	No	No	No	No	Yes	Yes	Yes	No	Yes	No
Dayie et al., 2018 [10]	Yes	Yes	Yes	Yes	Yes	No	Yes	No	No	Yes	Yes	Yes	No	Yes	No
Norris et al., 2003 [11]	Yes	Yes	Yes	Yes	Yes	Yes	Yes	No	No	Yes	Yes	No	Yes	Yes	No
Miller et al., 2005 [12]	Yes	Yes	Yes	Yes	No	No	No	No	No	No	Yes	No	No	Yes	No
Appiah et al., 2020 [13]	Yes	Yes	No	Yes	Yes	No	Yes	No	No	Yes	Yes	Yes	No	Yes	Yes
Mutagonda et al., 2022 [14]	Yes	Yes	Yes	Yes	Yes	No	Yes	No	No	Yes	Yes	Yes	No	Yes	No
Subudhi et al., 2021 [15]	Yes	Yes	Yes	Yes	Yes	No	Yes	No	No	Yes	Yes	Yes	No	Yes	Yes

Brown et al., 2003 [16]	Yes	Yes	No	Yes	Yes	No	No	No	No	No	Yes	Yes	No	Yes	No
Daw et al., 1997 [17]	Yes	Yes	No	Yes	Yes	No	No	No	No	Yes	Yes	Yes	No	Yes	No
Sangeda et al., 2024 [18]	Yes	Yes	Yes	Yes	Yes	No	Yes	No	Yes	Yes	Yes	Yes	No	Yes	Yes

References

1. Abdulmanea, A.A.; Alharbi, N.S.; Somily, A.M.; Khaled, J.M.; Algahtani, F.H. The Prevalence of the Virulence Genes of Staphylococcus aureus in Sickle Cell Disease Patients at KSUMC, Riyadh, Saudi Arabia. *Antibiotics* **2023**, *12*, 1221. https://doi.org/10.3390/antibiotics12071221.

2. Donkor, E.S.; Foster-Nyarko, E.; Enweronu-Laryea, C.C. Relationship between antibiotic resistance and sickle cell anemia: Preliminary evidence from a pediatric carriage study in Ghana. *Infect. Drug Resist.* **2013**, *6*, 71–77. https://doi.org/10.2147/IDR.S40062.

3. Dayie, N.T.; Sekoh, D.N.; Tetteh-Quarcoo, P.B.; Dayie, A.D.; Osei, M.-M.; Kotey, F.C.; Donkor, E.S. Staphylococcus aureus Nasopharyngeal Carriage and Antimicrobial Resistance among Adults with Sickle Cell Disease at the Korle Bu Teaching Hospital in Accra, Ghana. *Microbiol. Insights* **2022**, *15*, 11786361221133959. https://doi.org/10.1177/11786361221133959.

4. Dayie, N.T.K.D.; Sekoh, D.N.K.; Kotey, F.C.N.; Egyir, B.; Tetteh-Quarcoo, P.B.; Adutwum-Ofosu, K.K.; Ahenkorah, J.; Osei, M.-M.; Donkor, E.S. Nasopharyngeal Carriage of Methicillin-Resistant Staphylococcus aureus (MRSA) among Sickle Cell Disease (SCD) Children in the Pneumococcal Conjugate Vaccine Era. *Infect. Dis. Rep.* **2021**, *13*, 191–204. https://doi.org/10.3390/idr13010022.

5. Mava, Y.; Bello, M.; Ambe, J.P.; Zailani, S.B. Antimicrobial sensitivity pattern of organisms causing urinary tract infection in children with sickle cell anemia in Maiduguri, Nigeria. *Niger. J. Clin. Pract.* **2012**, *15*, 420–423. https://doi.org/10.4103/1119-3077.104515.

6. Lo, S.W.; Hawkins, P.A.; Jibir, B.; Hassan-Hanga, F.; Gambo, M.; Olaosebikan, R.; Olanipekun, G.; Munir, H.; Kocmich, N.; Rezac-Elgohary, A.; et al. Molecular characterization of streptococcus pneumoniae causing disease among children in nigeria during the introduction of pcv10 (Gsk). *Microb. Genomics* **2023**, *9*, 001094. https://doi.org/10.1099/mgen.0.001094.

7. Said, M.M.; Msanga, D.R.; Mtemisika, C.I.; Silago, V.; Mirambo, M.M.; Mshana, S.E. Extended Spectrum β-Lactamase Producing Lactose Fermenting Bacteria Colonizing Children with Human Immunodeficiency Virus, Sickle Cell Disease and Diabetes Mellitus in Mwanza City, Tanzania: A Cross-Sectional Study. *Trop. Med. Infect. Dis.* **2022**, *7*, 144. https://doi.org/10.3390/tropicalmed7080144.

8. Dibbasey, M.; Dahaba, M.; Sarfo, F.; Jallow-Manneh, I.; Ceesay, B.; Umukoro, S.; Diop, M.F.; Amambua-Ngwa, A. Laboratory indices of hospitalized sickle cell disease patients, prevalence and antimicrobial susceptibility of pathogenic bacterial isolates at MRCG ward in the Gambia. *BMC Infect. Dis.* **2023**, *23*, 546. https://doi.org/10.1186/s12879-023-08542-z.

9. Steele, R.W.; Warrier, R.; Unkel, P.J.; Foch, B.J.; Howes, R.F.; Shah, S.; Williams, K.; Moore, S.; Jue, S.J. Colonization with antibiotic-resistant Streptococcus pneumoniae in children with sickle cell disease. *J. Pediatr.* **1996**, *128*, 531–535. https://doi.org/10.1016/S0022-3476(96)70365-7.

10. Dayie, N.T.K.D.; Tetteh-Ocloo, G.; Labi, A.-K.; Olayemi, E.; Slotved, H.-C.; Lartey, M.; Donkor, E.S. Pneumococcal carriage among sickle cell disease patients in Accra, Ghana: Risk factors, serotypes and antibiotic resistance. *PLoS ONE* **2018**, *13*, e0206728. https://doi.org/10.1371/journal.pone.0206728.

11. Norris, C.F.; Smith-Whitley, K.; McGowan, K.L. Positive blood cultures in sickle cell disease: Time to positivity and clinical outcome. *J. Pediatr. Hematol. Oncol.* **2003**, *25*, 390–395. https://doi.org/10.1097/00043426-200305000-00008.

12. Miller, M.L.; Obert, C.A.; Gao, G.; Daw, N.C.; Flynn, P.; Tuomanen, E. Cephalosporin-resistant Pneumococci and sickle cell disease. *Emerg. Infect. Dis.* **2005**, *11*, 1192–1196. https://doi.org/10.3201/eid1108.050152.

13. Appiah, V.A.; Pesewu, G.A.; Kotey, F.C.N.; Boakye, A.N.; Duodu, S.; Tette, E.M.A.; Nyarko, M.Y.; Donkor, E.S. Staphylococcus aureus nasal colonization among children with sickle cell disease at the children's hospital, accra: Prevalence, risk factors, and antibiotic resistance. *Pathogens* **2020**, *9*, 329. https://doi.org/10.3390/pathogens9050329.

14. Mutagonda, R.F.; Bwire, G.; Sangeda, R.Z.; Kilonzi, M.; Mlyuka, H.; Ndunguru, J.; Jonathan, A.; Makani, J.; Minja, I.K.; Ruggajo, P.; et al. Nasopharyngeal Carriage and Antibiogram of Pneumococcal and Other Bacterial Pathogens from Children with Sickle Cell Disease in Tanzania. *Infect. Drug Resist.* **2022**, *15*, 4407–4418. https://doi.org/10.2147/IDR.S367873.

15. Subudhi, M.; Jagatheeswary, P.A.T.; Sahu, S.K.; Das, S.K.; Subudhi, K.B.; Rout, R.R. Incidence and variation of microbiological profile of catheter-associated urinary tract infection in precise comorbidities associated with tribal sickle cell anemic patients of medical intensive care unit in a tribal tertiary care center. *J. Appl. Hematol.* **2021**, *12*, 140–146. https://doi.org/10.4103/joah.joah_184_20.

16. Brown, B.J.; Asinobi, A.O.; Fatunde, O.J.; Osinusi, K.; Fasina, N.A. Antimicrobial sensitivity pattern of organisms causing urinary tract infection in children with sickle cell anaemia in Ibadan, Nigeria. *West Afr. J. Med.* **2003**, *22*, 110–113.

17. Daw, N.C.; Wilimas, J.A.; Wang, W.C.; Presbury, G.J.; Joyner, R.E.; Harris, S.C.; Davis, Y.; Chen, G.; Joan Chesney, P. Nasopharyngeal carriage of penicillin-resistant streptococcus pneumoniae in children with sickle cell disease. *Pediatrics* **1997**, *99*, 594–595. https://doi.org/10.1542/peds.99.4.e7.

18. Sangeda, R.; Yohana, J.; Jonathan, A.; Manyanga, V.; Soka, D.; Makani, J. Prevalence of Urinary Tract Infections and Antibiogram of Bacteria Isolated From Children With Sickle Cell Disease in Tanzania. *CUREUS J. Med. Sci.* **2024**, *16*, e58786. https://doi.org/10.7759/cureus.58786.
